# Artificial intelligence and machine learning in heart and lung transplantation

**DOI:** 10.1016/j.eclinm.2026.104180

**Published:** 2026-09-03

**Authors:** Gaurav Sharma, Vineet Kumar Kamal, Lauren K. Truby, Maryjane Farr, Irina Timofte, Vaidehi Kaza, Srdjan Lesaja, John S.K. Murala, Suresh Keshavamurthy, Matthias Peltz, Michael E. Jessen

**Affiliations:** a Department of Cardiovascular and Thoracic Surgery, University of Texas Southwestern Medical Center, Dallas, TX, USA; b Advanced Imaging Research Center, University of Texas Southwestern Medical Center, Dallas, TX, USA; c Department of Biomedical Engineering, University of Texas Southwestern Medical Center, Dallas, TX, USA; d Department of Biostatistics, All India Institute of Medical Sciences, Kalyani, India; e Division of Cardiology, Department of Medicine, University of Texas Southwestern Medical Center, Dallas, TX, USA; f Penn Heart and Vascular Center, University of Pennsylvania Health System, Philadelphia, PA, USA; g Division of Pulmonary and Critical Care Medicine, Department of Internal Medicine, University of Texas Southwestern Medical Center, Dallas, TX, USA; h Department of Research, United Network for Organ Sharing, Richmond, VA, USA

**Keywords:** Artificial intelligence, Machine learning, Heart transplantation, Lung transplantation, Organ allocation, Digital pathology

## Abstract

Artificial intelligence (AI) and machine learning (ML) are increasingly applied across the heart and lung transplant continuum, from donor organ evaluation and waitlist prioritisation through peri-operative decision support to post-transplant rejection surveillance and personalised immunosuppression. Deep learning for endomyocardial biopsy interpretation has achieved an area under the curve (AUC) of 0.962, with performance comparable to expert pathologists, while ML-augmented ex-vivo lung perfusion platforms have demonstrated the potential to expand organ utilisation in real-world implementation studies. Noninvasive donor-derived cell-free DNA assays and electrocardiogram-based deep learning models are beginning to reduce dependence on invasive surveillance biopsies. Across published studies, models predicting 1-year post-transplant mortality have shown only moderate discrimination. Most published models remain retrospectively validated on single-centre datasets with limited external validation, inconsistent calibration reporting, and unresolved concerns about algorithmic bias and global health equity. This scoping review synthesises current evidence, critically appraises model performance, and identifies the regulatory, ethical, and methodological steps required for responsible clinical integration. Future research should prioritise prospective, multicentre external validation, transparent calibration reporting, and formal evaluation of clinical utility before these tools are adopted in routine transplant care.

**Search strategy and selection criteria**

For this scoping review, we searched PubMed, MEDLINE, Embase, Web of Science for articles published between Jan 1, 2015, and May 31, 2026, using combinations of initial search terms including “artificial intelligence”, “machine learning”, “deep learning”, “neural network”, “heart transplantation”, “lung transplantation”, and subsequently added additional keywords “thoracic transplantation”, “organ allocation”, “rejection”, “graft dysfunction”, “immunosuppression”, and “digital pathology”. Records were screened by two reviewers, with disagreements resolved by discussion. We also reviewed reference lists of identified systematic reviews and meta-analyses. The start date of Jan 1, 2015, reflects the emergence of modern machine-learning and deep-learning methods in clinical prediction, and the end date of May 31, 2026, reflects the most recent literature available when the search was run. We prioritised peer-reviewed studies reporting quantitative performance metrics, prospective validation cohorts, multi-centre designs, and systematic reviews with meta-analyses. Conference abstracts were included only when they represented the sole available evidence for a specific application. As a Review of published studies, the authors did not collect individual participant-level race or ethnicity data; representativeness and the structural drivers of any race-associated differences are considered in the section on algorithmic bias and health equity. The final reference list was generated based on originality and relevance to the broad scope of this Review. An accompanying meta-analysis was conducted. Full details are given within the Supplementary data.

## Introduction

Thoracic organ transplantation has undergone remarkable growth over the past decade. The International Society for Heart and Lung Transplantation (ISHLT) Thoracic Organ Transplant Registry includes data from 182,319 transplant recipients between 1992 and 2024 across 12 global partners.^1^ Since 2013, annual heart transplant volume has increased progressively, reaching 5887 in 2023 among contributing centres worldwide.^2^^,^^3^ Meanwhile, global lung transplant volumes have gradually recovered following a pandemic-related decline from their 2019 peak of around 3800 annual procedures. However, this recovery in lung volume is currently counterbalanced by a sharp reduction in transplants for cystic fibrosis patients due to advances in targeted therapeutics.^1^ Despite these achievements, the gap between organ supply and demand remains vast. Approximately 50,000 candidates await heart transplantation globally, yet only about 30% of procured donor hearts are used for transplantation.^1^ Lung utilisation rates have historically ranged between 15% and 20%, though more recent estimates suggest improvement with expanded donor criteria protocols and organ preservation technology.^4^

**Fig. 1:**
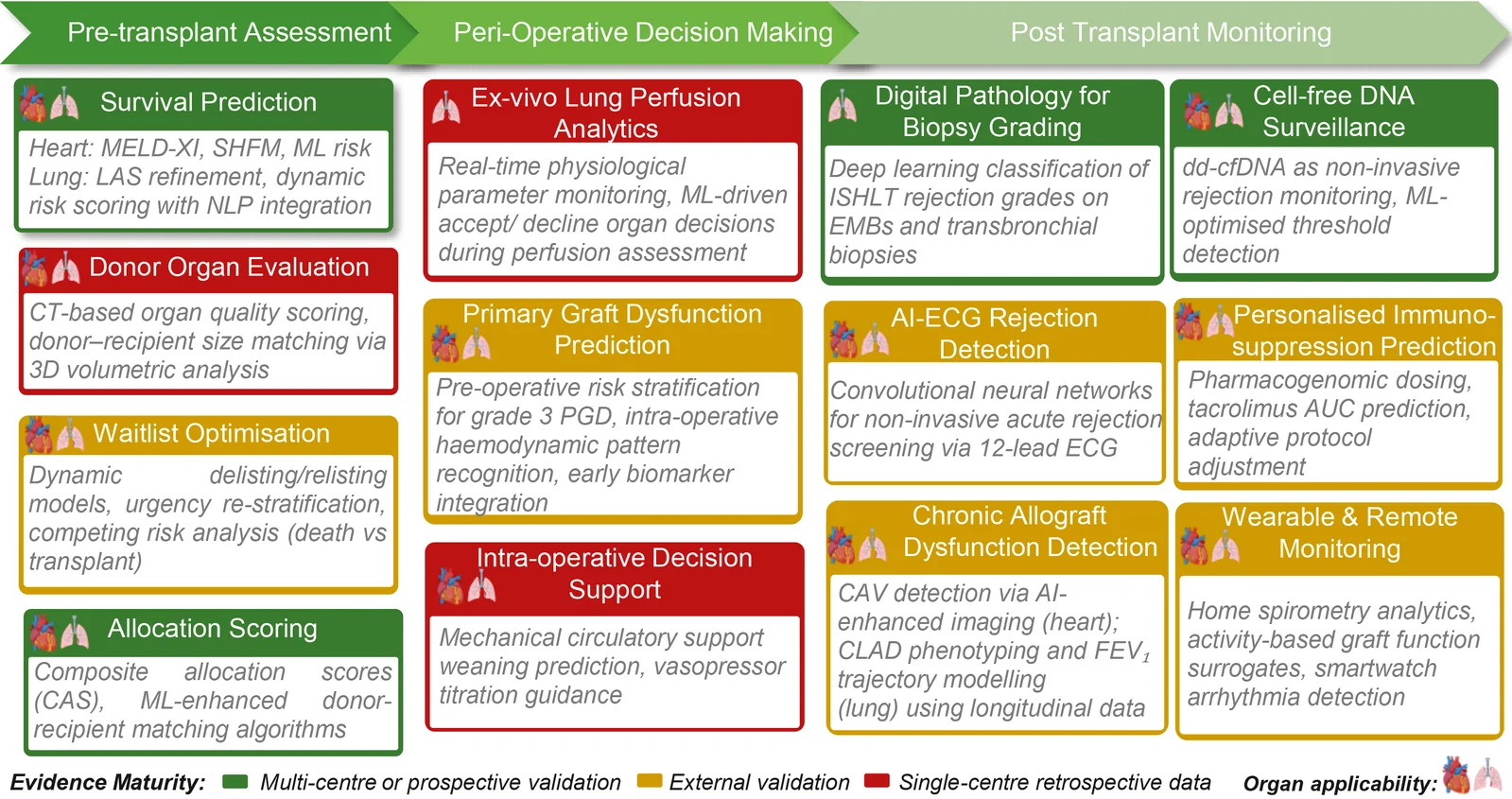
**Artificial intelligence and machine learning applications across the heart and lung transplant continuum**. The schematic outlines algorithmic integration throughout pre-transplant assessment, peri-operative decision-making, and post-transplant monitoring phases. Colour coding denotes the maturity of clinical evidence: green indicates multi-centre or prospective validation, amber signifies external validation, and red represents applications limited to single-centre retrospective data. Abbreviations: AI = artificial intelligence; CAS = composite allocation score; CAV = cardiac allograft vasculopathy; CLAD = chronic lung allograft dysfunction; CT = computed tomography; dd-cfDNA = donor-derived cell-free DNA; ECG = electrocardiogram; EMB = endomyocardial biopsy; EVLP = ex-vivo lung perfusion; FEV1 = forced expiratory volume in 1 second; ISHLT = International Society for Heart and Lung Transplantation; LAS = lung allocation score; ML = machine learning; MELD-XI = Model for End-Stage Liver Disease excluding INR; NLP = natural language processing; PGD = primary graft dysfunction; SHFM = Seattle Heart Failure Model.

Clinical decision-making in transplantation is uniquely complex (Fig. 1). Clinicians must weigh recipient acuity against donor organ quality, immunological compatibility, and logistical constraints, often under severe time pressure. Traditional risk-scoring systems, including the Index for Mortality Prediction After Cardiac Transplantation (IMPACT), the Donor Risk Index, and the Lung Allocation Score (LAS), rely on a limited number of linear predictors and typically yield C-statistics of approximately 0.61, while lung allocation models achieve only 0.55–0.62 for posttransplant survival prediction.^5^, ^6^, ^7^ The recently implemented Composite Allocation Score (CAS) for lung transplantation, which replaced the LAS in March 2023, incorporates post-transplant survival alongside medical urgency, but early outcome data remain limited and the system's long-term impact on allocation equity requires further evaluation.^8^ Artificial intelligence (AI) and machine learning (ML) offer the potential to move beyond these constraints by modelling nonlinear relationships among hundreds of clinical, imaging, genomic, and molecular variables. Over the past decade, a rapidly expanding literature has applied techniques from gradient-boosted decision trees and random survival forests to convolutional neural networks and attention-based deep learning across virtually every phase of the transplant journey.^9^, ^10^, ^11^ The pace of development, however, has outpaced rigorous validation. Many published studies remain single centre, retrospectively designed, and lacking in external validation or prospective clinical testing. The clinical stakes in transplantation are exceptionally high: allocation decisions are irreversible, organ wastage carries population-level consequences, and errors in rejection surveillance can result in graft loss or unnecessary immunosuppression with attendant toxicity. These characteristics demand a standard of evidence that exceeds what is typically required for AI deployment in lower-stakes clinical domains. This imbalance between technological enthusiasm and evidentiary maturity frames the central tension of this review.

This scoping review aims to synthesise current evidence across the full transplant continuum, evaluate specific model performance metrics with attention to validation rigour, and identify critical methodological, regulatory, and ethical gaps that must be addressed before AI can be responsibly embedded in thoracic transplant practice (Table 1). An accompanying meta-analysis of machine-learning models predicting 1-year post-transplant mortality was undertaken to quantify discrimination across published heart and lung transplant cohorts and is reported in accordance with the Preferred Reporting Items for Systematic Reviews and Meta-Analyses (PRISMA) 2020 statement and its extension for Scoping Reviews (PRISMA-ScR). The full meta-analytic methods, together with the PRISMA 2020 (Supplementary Figure S1), PRISMA-ScR (Supplementary Figure S2) flow diagrams and meta-analysis (Supplementary Figure S3), are provided in Supplementary data.

**Table 1:** Machine learning and artificial intelligence applications in heart and lung transplantation: Algorithms, datasets, performance metrics, and validation methods.

Application	Study	Algorithm/model	Dataset (N)	Performance metric(s)	Key findings/outcome	Validation method
Heart transplant (HTx)						
HTx—Survival and Mortality Prediction						
Heart transplant survival	Nilsson J et al. (2015)^5^	Neural network (IHTSA)	Developed on 41,780; total 56,625; internal + temporal + external validation	AUC 0.650; C-index 0.600 [95% CI: 0.595–0.604]	Prediction of 1-year mortality post-heart transplantation using IHTSA vs IMPACT risk models	External
Heart transplant 1-yr mortality	Miller PE et al. (2019)^12^	LR, SVM, RF, DT, NN	56,477	AUC: logistic regression 0.66 Ridge Regression 0.65 Regression with LASSO 0.65 Neural Network 0.66 SVM 0.52 Naïve Bayes 0.61 TAN-Bayes 0.62 Random Forest 0.63 Boosted Trees 0.64	Modest discrimination for 1-year mortality; ML comparable to traditional regression	Internal (cross-validation)
Heart transplant 5-yr mortality/graft failure	Agasthi P et al. (2020)^13^	Gradient boosted machine	15,236	AUC 0.717 (mortality); 0.716 (graft failure)	Prediction of 5-year mortality and graft failure; top 10 influential variables identified	Internal (10-fold CV)
Heart transplant 1-yr mortality	Kampaktsis PN et al. (2021)^14^	AdaBoost, SVM, DT, KNN, LR	18,625	AUC: AdaBoost 0.689 LR 0.642 Decision Tree 0.649 SVM 0.637 k-nearest neighbour 0.526	1-year post-transplant mortality risk prediction	Internal (cross-validation)
Heart transplant 1-yr mortality	Ayers B et al. (2021)^15^	Ensemble (DNN + RF + AdaBoost + LR)	33,657	AUC 0.764 (ensemble); Neural Network 0.691; Logistic Regression 0.691; Random Forest 0.691; AdaBoost 0.653	Ensemble ML outperformed traditional risk models for 1-year mortality	Internal (train/test split)
Heart transplant survival probabilities	Dolatsara HA et al. (2020)^16^	LR, XGB, LDA, RF, ANN, CART	103,570	AUC 0.60–0.71 (years 1–10); LR 0.651; XGB 0.649; LDA 0.648; RF 0.649; ANN 0.616; CART 0.599 (1-year timeframe)	Calibrated survival probabilities over years 1–10 using isotonic regression	Internal (isotonic calibration)
Heart transplant 3-mo to 10-yr mortality	Yoon J et al. (2018)^17^	Trees of Predictors (ToPs/R)	51,971	AUC 0.660 (3-month); 0.641 (1-year); 0.623 (3-year); 0.631 (5-year); C-index 0.577	Personalised survival prediction; ToP outperformed clinical risk scores at 3-month to 10-year	Internal (cross-validation)
Heart transplant 1-yr mortality	Zhou Y et al. (2021)^18^	RF, AdaBoost, LR, SVM, XGBoost, GBM, ANN	381	AUC 0.801 (RF best); XGBoost 0.769; GBM 0.786; ANN 0.755; SVM 0.714; LR 0.688; AdaBoost 0.641; naïve Bayes 0.500	Short-term 1-year mortality; top 5 predictors: albumin, age, LA diameter	Internal (single centre)
Paediatric heart transplant 1,3,5-yr mortality	Miller R et al. (2019)^19^	ANN, CART, RF	2802	AUC 0.72 (1-yr); 0.61 (3-yr); 0.60 (5-yr)	Prediction of 1-, 3-, and 5-year mortality in paediatric heart transplant recipients	Internal (train/test split)
Paediatric heart transplant graft failure (1,2-yr)	Altamimi O et al. (2026)^20^	Random Survival Forest (RSF); scikit-survival implementation	UNOS registry post-July 2016; n = 4162 (train 3121; test 1041)	C-index 0.60; 1-yr AUC 0.636; 2-yr AUC 0.630; 1-yr Brier score 0.078; DCA net benefit confirmed	RSF stratifies paediatric HTx graft-failure risk into low- vs high-risk groups (1-yr survival 92.8% vs 88.1%; log-rank p = 0.003)	Internal (75:25 random split; no external validation)
Heart transplant survival	Lisboa PJG et al. (2022)^21^	Self-explaining NN vs DL (IHTSA)	4750	AUC 0.628 (PRN-Lasso)	Improved prediction and model interpretability using deep learning vs traditional NN	External (2 cohorts)
Heart transplant survival	Mohammadi I et al. (2025)^22^	Multiple ML models pooled	12 studies pooled	Pooled AUC 0.65; CatBoost accuracy AUC 0.80	Pooled ML performance for post-transplant survival prediction; modest overall discrimination	Mixed
Heart transplant 1-yr mortality—interpretable multicentre	Zhou X et al. (2026)^23^	GINN (Generalisable Interpretable Neural Network); additive representation	UNOS 1994–2024, Eurotransplant, Scandiatransplant registries, Jiangxi cohort (CN); total N not reported	AUROC: UNOS 0.827; Eurotransplant 0.789; Scandiatransplant 0.776; External-CN 0.821	Interpretable neural network (nine clinical variables) with cross-registry external validation of HTx mortality across four geographically distinct cohorts; key predictors: donor/recipient age, donor function, pre-op support, diagnosis type.	External (4 independent cohorts across UNOS, EU, Scandinavia, China)
HTx—Waitlist Mortality and Allocation						
Waitlist mortality prediction	Hsich EM et al. (2019)^24^	Random survival forest	33,069	Key variables identified; eGFR and albumin additive	Identified key predictors (eGFR, albumin) of waitlist mortality not in current allocation system	Internal (variable importance)
Waitlist outcome prediction	Medved D et al. (2017)^25^	ANN	27,444	Top 10 predictive variables weighted	Prediction of waitlist outcome (waiting, transplanted, or dead) at 180, 365, 730 days	Internal
Allocation simulation	Medved D et al. (2018)^26^	NN (IHTSA + LuDeLTA)	49,566	NN allocation: ∼4700 days vs 4300 days (wait time)	NN-based allocation improved predicted mean survival vs clinical rules and waitlist-time allocation	Internal (simulation)
HTx—Rejection and Allograft Monitoring						
Cardiac rejection (endomyocardial biopsy)	Tong L et al. (2017)^27^	Deep neural network	43	High accuracy vs manual grading	Automated grading of rejection in whole-slide biopsy images; reduced overfitting with regularisation	Internal
Cardiac rejection (biopsy)	Lipkova J et al. (2022)^28^	Attention-based DL (CRANE)	Multi-centre (US, Turkey, Switzerland)	AUC 0.962 (rejection)	AI-assisted grading of endomyocardial biopsy whole-slide images	External
Molecular rejection (cardiac)	Halloran PF et al. (2024)^29^	Ensemble ML (MMDx)	3230 endomyocardial biopsies (1641 from the INTERHEART trial + 1589 clinical service biopsies).	Seven molecular rejection archetypes; frequent molecular and histologic discordance	Six injury-independent classifiers defined seven rejection archetypes; many histologic rejection diagnoses mapped to no-rejection or minor molecular states, and parenchymal injury, more than AMR, was associated with graft loss	Multi-centre
Molecular vs histologic rejection (cardiac, real-world)	Alam A et al. (2022)^30^	MMDx vs endomyocardial biopsy	228 paired specimens; 135 recipients	84% agreement; Cohen's kappa 0.48 (p < 0.001)	Real-world comparison of MMDx with histology; MMDx identified more rejection events than histology alone	Single-centre, real-world
Non-invasive rejection (dd-cfDNA)	Khush K et al. (2019)^31^	Bioinformatic (AlloSure)	740 recipients (26 US centres)	Sensitivity 44%; NPV 97%	Donor-derived cell-free DNA for non-invasive rejection surveillance with high negative predictive value	Prospective
dd-cfDNA cardiac rejection surveillance (FreeDNA-CAR trial)	Jiménez-Blanco M et al. (2025)^32^	Biomarker study-Allonext NGS dd-cfDNA assay; combined dd-cfDNA + NT-proBNP model (logistic)	206 recipients; 12 Spanish centres; 1090 paired EMB/dd-cfDNA determinations; NCT04973943	dd-cfDNA alone AUC 0.603 for ACR; dd-cfDNA + NT-proBNP AUC 0.681 (p = 0.016 vs dd-cfDNA alone); NPV 97% at 0.10% threshold; NPV 98.1% with combined biomarker approach	Prospective European multicentre dd-cfDNA study: dd-cfDNA alone has modest discrimination for ACR but high NPV; adding NT-proBNP substantially improves discrimination.	Prospective multicentre (12 centres, Spain)
dd-cfDNA vs EMB—interrater reliability and long-term outcomes (GRAfT)	Mehta A et al. (2025)^33^	Bioinformatic (AlloSure dd-cfDNA); comparative biomarker study vs histopathology	GRAfT registry; 94 patients; 429 paired EMB/dd-cfDNA measures; multicentre prospective (2015–2020)	Pathologist concordance: ACR 77% (kappa moderate); AMR 63% (lower); dd-cfDNA LR+ 3.74 (centre path.) vs 2.59 (core path.) for AR; LR + improves to 9.82 when longitudinal composite outcomes included	dd-cfDNA predictive value for clinical outcomes (rejection, allograft dysfunction, 1-yr mortality) exceeds its performance against histopathological rejection alone; also quantifies AMR interrater variability in multicentre data.	Prospective multicentre (GRAfT consortium)
Allograft rejection detection (AI-ECG)	Adedinsewo D et al. (2023)^34^	CNN (12-lead ECG)	7590	AUC 0.84; Sensitivity 95%; Specificity 52.6%	Detection of allograft rejection from routine 12-lead ECG using CNN	Prospective
Extracellular vesicle surface antigen profiling for ACR detection	Burrello J et al. (2025)^35^	Random forest regressor; adaptive patient-specific AI model; flow cytometry-derived EV surface antigens	Prospective longitudinal; 24 HTx recipients; 285 blood samples; median follow-up 303 days; independent validation cohort	LOOCV AUC 0.968; accuracy 93.3%; Specificity 95%; NPV 96.2%; Independent cohort AUC 0.832; accuracy 78.9%	Prospective study: EV antigen profiling with patient-specific adaptive AI detects ACR before histological diagnosis (NPV 96.2%); limited by small sample (n = 24; 285 samples), with lower independent-cohort performance (AUC 0.832).	Internal LOOCV + independent cohort validation
HTx—Immunosuppression and Vasculopathy						
CAV phenotyping	Moayedi Y et al. (2024)^36^	Latent class IVUS analysis	186	4 clusters; risk 0–49.1%	Identification of 4 cardiac allograft vasculopathy phenotypic clusters with distinct risk profiles	Internal
Cardiac PGD prediction	Ding S et al. (2025)^37^	Five ML models; multi-layer perceptron (MLP); explainable AI	8008 (OPTN)	AUROC 0.868 (MLP); top-20 donor–recipient features identified	MLP outperformed other models for cardiac PGD; explainable AI surfaced key donor–recipient interactions	Internal
Cardiac PGD/90-day graft failure	Yim WY et al. (2026)^38^	XGBoost-informed nomogram; eight donor/recipient predictors	25,200 (OPTN, 2008–2020)	AUC 0.67 (95% CI 0.64–0.70); external Chinese cohort AUC 0.67; 2.4-fold hazard in highest-risk stratum	Nomogram stratified 90-day graft-failure risk; highest-risk stratum had 2.4-fold hazard	External
**Lung transplant (LTx)**						
LTx—Survival and Mortality Prediction						
Lung transplant survival (1,5,10-yr)	Sharma G et al. (2025)^39^	AutoScore-Survival (RSF + Cox hybrid)	51,933	iAUC 0.61; time-dependent AUC 0.61 (1-yr), 0.59 (5-yr), 0.72 (10-yr); C-index 0.64	Interpretable 9-variable risk score for 1-, 5-, 10-year post-transplant survival	Temporal validation (2015–2025)
Lung transplant overall survival	Tian D et al. (2023)^40^	Random survival forest	504	iAUC 0.879 (95% CI 0.832–0.921); Cox iAUC 0.658	Overall survival prediction; RSF outperformed Cox regression	Internal (bootstrap)
Lung transplant survival	Yeo HJ et al. (2025)^41^	Gradient boosting, LR, SVM, RF, MLP, BRF	29,364	GBM internal AUC 0.958; GBM external AUC 0.852; MLP best external generalisation AUC 0.911	1-year post-transplant survival prediction using international registry data	Internal + external
Lung transplant 1,5,10-yr survival	Zafar F et al. (2022)^42^	Cox-LASSO regression	15,124	C-statistic: 1-yr 0.67; 5-yr 0.64; 10-yr 0.72	Survival prediction and risk cluster stratification at 1, 5, and 10 years	Internal (train/test split)
Lung transplant 1,3-yr mortality	Brahmbhatt JM et al. (2022)^43^	LAS, LASSO, RF	19,900	LASSO AUC 0.61; RF AUC 0.62; LAS AUC 0.55; overall AUC 0.55–0.62; NPV 0.87–0.90	Post-transplant mortality; ML models showed limited improvement over LAS	Internal (train/test split)
Lung transplant 1,5,10-yr survival	Moro A et al. (2024)^44^	Logistic regression + DT subgroups	27,296	C-index 0.653	Survival probability estimation with 8 DT-derived risk subgroups	Internal (cross-validation)
Prolonged lung transplant survival	Amini M et al. (2022)^45^	RF, DT, GBT, KNN, ANN, SVM, LR	13,080	AUC 0.79 (RF best); Accuracy 77.9%; GBT 0.74; LR 0.75	Factors predicting prolonged graft survival; RF identified key explanatory variables	Internal (10-fold CV)
Lung transplant 5-yr survival (IRD)	Mark E et al. (2019)^46^	Cox proportional hazards, RSF	UNOS (IRD n = 1010; non-IRD n = 12,013)	RMSE 5.3; 7.2% 5-year survival benefit with IRD	Survival benefit of increased-risk donor organs vs standard; 7.2% 5-year benefit	Internal (cross-validation)
LTx—Waitlist and Organ Allocation						
Waitlist and post-transplant survival	Dalton JE et al. (2023)^7^	RSF, GBT, logistic, LDA, CAS models	SRTR (WL n = 13,204; Tx n = 20,763)	WL AUC 0.85–0.93; PT AUC 0.56–0.62	Refinement of lung allocation scoring; ML-based waitlist models vastly outperformed post-transplant models	Internal (10-fold CV)
6-month graft survival (allocation)	Dueñas-Jurado JM et al. (2021)^47^	Logistic regression + product units	404	FVC and FEV1 key predictors identified	Donor-recipient matching optimisation; FVC and FEV1 identified as key graft survival predictors	Internal (train/test split)
LTx—Donor Lung Assessment and EVLP						
Donor lung CT assessment	Ram S et al. (2024)^48^	Dictionary learning (CT-ML)	100	AUC 0.89; 19-fold CLAD risk for ML-declined lungs	CT-based donor lung screening; ML-declined lungs had 19-fold increased CLAD risk at 2 years	Internal (prospective)
EVLP decision support	Sage AT et al. (2023)^49^	XGBoost (InsighTx)	725	AUROC 0.90 (unsuitable lungs); 0.80 (good outcomes); training 0.79 ± 0.03; independent test 0.75 ± 0.04 and 0.85 ± 0.03	AI-driven EVLP decision tool; AUROC 0.90 for identifying unsuitable lungs, influenced clinical decisions	Blinded retrospective
EVLP multimodal assessment	Chao BT et al. (2024)^50^	ResNet-50 radiographic features + XGBoost	1300 radiographs (650 EVLP cases)	AUROC 0.94 (vs 0.88 for InsighTx alone; p < 0.0001)	Adding CNN radiographic features to InsighTx improved donor-lung outcome classification (accuracy 73%–78%, AUROC 0.88 to 0.94)	Internal (validation set)
Donor lung size prediction	Pu L et al. (2023)^51^	U-Net + MLP/XGBoost	4610	Dice 0.951 (right lung); R^2^ 0.628 (MLP)	Automated lung volume estimation from CT for donor-recipient size matching	Internal (10-fold CV)
Automated organ-donor identification	Sauthier N et al. (2023)^52^	Temporal deep neural network; routine ICU data	Multicentre ICU cohort	AUC 0.966	Identified potential donors missed by medical teams and manual death audits	Internal
LTx—Primary Graft Dysfunction (PGD) Prediction						
PGD prediction (lung)	Diamond JM et al. (2024)^53^	Regularised logistic regression	Multicentre prospective	AUC 0.76 (C-statistic)	Validated PGD risk prediction model for clinical decision support	External
PGD grade 3 prediction within 72 h	Michelson AP et al. (2024)^54^	LR, KNN, XGBoost, SVC	576	AUROC: KNN 0.65; XGBoost 0.72; SVC 0.55	Pretransplant prediction of severe PGD (grade 3) within 72 h; open-access risk calculator	Internal (5-fold CV)
PGD grade 3 prediction (staged)	Fessler J et al. (2025)^55^	XGBoost, LR, SHAP	477	AUROC 0.84 (XGBoost, final stage); LR 0.81	Progressive PGD grade 3 prediction across 9 transplant stages with SHAP interpretability	Internal (train/test split, 5-fold CV)
PGD grade 3 prediction at 72 h	Xia W et al. (2025)^56^	RF, DT, KNN, MLP, SVM (9 models)	802	AUC 0.80 (internal validation); 0.821 (external validation cohort)	PGD grade 3 classification; blood loss identified as key predictor via SHAP	Internal + external (3-way split + 5-fold CV; external validation cohort)
PGD risk (donor lung genomics)	Cantu E et al. (2020)^57^	Deep learning (TLR pathways)	113	AUC 0.776; Sensitivity 0.786; NPV 0.910	Genomic-based PGD risk prediction using TLR pathway gene expression from donor lung biopsies	Internal (5-fold CV)
LTx—Rejection and Chronic Lung Allograft Dysfunction (CLAD)						
BOS detection (CT)	Kozinski M et al. (2025)^58^	Deep neural network	Multi-centre	AUC 0.90	Automated BOS detection from CT imaging using deep learning	Internal
BOS classification (CT)	Barbosa EM et al. (2017)^59^	SVM with qCT	176	AUC 0.817 (unilateral); 0.765 (bilateral)	BOS diagnosis using quantitative CT features; superior for unilateral lung recipients	Internal (jackknife CV)
BOS classification (3D CT)	Barbosa EJM et al. (2018)^60^	SVM (functional respiratory imaging)	71	Accuracy 85%; Sensitivity 73.3%; Specificity 92.3%	3D functional respiratory imaging with SVM for BOS classification	Internal (cross-validation)
CLAD phenotyping	McInnis MC et al. (2022)^61^	CALIPER (ANN, Bayes, SVM, KNN)	88	AUC 0.851; Sensitivity 0.90; Specificity 0.71	Automated CLAD phenotyping and prognostication using CALIPER CT analysis	Internal
CLAD identification (BAL peptides)	Berra G et al. (2021)^62^	SVM (Ang II peptides)	40	AUC 0.86 (CLAD vs stable); 0.97 (predict CLAD)	CLAD identification and future CLAD risk prediction using angiotensin II-related BAL peptides	Internal (LOOCV)
CLAD biomarkers (eNose)	Wijbenga N et al. (2023)^63^	Supervised ML (eNose sensor data)	152	AUC 0.94; Sensitivity 96–100%	Non-invasive CLAD detection via electronic nose; high sensitivity across phenotypes and stages	Internal (10-fold CV)
Lung transplant rejection (molecular)	Halloran KM et al. (2019),^64^ Halloran K et al. (2020)^65^	Unsupervised ML (microarray)	242 (TBB)^64^; 243 (mucosal)^65^	Molecular phenotyping of rejection subtypes	Molecular classification of lung transplant rejection using microarray-based transcriptomics	Multi-centre consensus
Tacrolimus dose prediction (lung)	Choshi H et al. (2025)^66^	Multivariate long short-term memory (LSTM) time-series	87,112 data points (117 patients)	Predictions within 30% of actual trough at 88.5% of timepoints	LSTM forecast tacrolimus troughs from longitudinal dose–concentration data; enabled dose–response simulation	Internal
Remote home spirometry monitoring (lung)	Odisho A et al. (2021)^67^	Chatbot-based mobile health intervention; home spirometry	Lung transplant recipients (remote-monitoring cohort)	Correlation r = 0.93 with laboratory-measured FEV1	Chatbot home spirometry correlated strongly with laboratory FEV1; alert algorithms flagged clinically significant decline	Prospective implementation
Federated multi-institutional learning	Sheller MJ et al. (2020)^68^	Federated deep learning (model-parameter exchange)	10 institutions (multi-institutional)	99% of centrally-pooled model performance	Privacy-preserving training across institutions without sharing patient data; near-parity with centrally pooled models	Multi-institutional (federated)

Studies are grouped by transplant type (heart transplant [HTx] and lung transplant [LTx]) and clinical domain, including survival and mortality prediction, waitlist management and organ allocation, rejection and allograft monitoring, immunosuppression optimisation, donor organ assessment, primary graft dysfunction prediction, and chronic allograft dysfunction. Performance metrics are reported as published; where multiple models were evaluated, the best-performing result is highlighted with individual model values listed. Validation approaches range from internal cross-validation to external and multicentre cohorts.

ACR = acute cellular rejection. AI = artificial intelligence. AI-ECG = artificial intelligence-based electrocardiogram model. AMR = antibody-mediated rejection. ANN = artificial neural network. AR = acute rejection. AUC = area under the curve. AUROC = area under the receiver operating characteristic curve. BAL = bronchoalveolar lavage. BOS = bronchiolitis obliterans syndrome. BRF = balanced random forest. CALIPER = Computer-Aided Lung Informatics for Pathology Evaluation and Rating. CART = classification and regression tree. CAS = composite allocation score. CAV = cardiac allograft vasculopathy. CLAD = chronic lung allograft dysfunction. CNN = convolutional neural network. CRANE = Cardiac Rejection Assessment Neural Estimator. CT = computed tomography. CV = cross-validation. DCA = decision curve analysis. dd-cfDNA = donor-derived cell-free DNA. DL = deep learning. DNN = deep neural network. DT = decision tree. ECG = electrocardiogram. eGFR = estimated glomerular filtration rate. EMB = endomyocardial biopsy. EV = extracellular vesicle. EVLP = ex-vivo lung perfusion. FEV1 = forced expiratory volume in 1 s. FVC = forced vital capacity. GBM = gradient boosted machine. GBT = gradient boosted trees. GINN = generalisable interpretable neural network. iAUC = integrated area under the curve. ICU = intensive care unit. IHTSA = International Heart Transplant Survival Algorithm. IMPACT = Index for Mortality Prediction After Cardiac Transplantation. IRD = increased risk for disease transmission. IVUS = intravascular ultrasound. KNN = k-nearest neighbour. LA = left atrial. LAS = lung allocation score. LASSO = least absolute shrinkage and selection operator. LDA = linear discriminant analysis. LOOCV = leave-one-out cross-validation. LR = logistic regression. LR+ = positive likelihood ratio. LSTM = long short-term memory. ML = machine learning. MLP = multi-layer perceptron. MMDx = Molecular Microscope Diagnostic System. NGS = next-generation sequencing. NN = neural network. NPV = negative predictive value. NT-proBNP = N-terminal pro-B-type natriuretic peptide. OPTN = Organ Procurement and Transplantation Network. PGD = primary graft dysfunction. PRN = partial response network. PT = post-transplant. qCT = quantitative computed tomography. RF = random forest. RMSE = root mean squared error. RSF = random survival forest. SHAP = Shapley Additive Explanations. SRTR = Scientific Registry of Transplant Recipients. SVC = support vector classifier. SVM = support vector machine. TAN = tree-augmented naïve Bayes. TBB = transbronchial biopsy. TLR = toll-like receptor. ToP = Trees of Predictors. UNOS = United Network for Organ Sharing. WL = waitlist. XGB/XGBoost = extreme gradient boosting.

Performance metrics are point estimates with 95% confidence intervals where these were reported in the source study.

## AI in the pre-transplant phase

### Survival prediction and donor–recipient matching

The prediction of post-transplant survival is central to organ allocation policy in some countries, although notably the current US heart allocation system prioritises medical urgency and waitlist status rather than predicted post-transplant survival. The International Heart Transplant Survival Algorithm (IHTSA), developed on 41,780 recipients and validated in an overall registry cohort of 56,625 recipients from the ISHLT registry, achieved a 1-year mortality area under the curve (AUC) of 0.650, compared with 0.61 for IMPACT.^5^^,^^6^ Medved et al. subsequently validated IHTSA on 27,860 transplants from the United Network for Organ Sharing (UNOS)-maintained Organ Procurement and Transplantation Network (OPTN) registry, and confirmed a sustained advantage (AUC 0.654 vs 0.608 for IMPACT; p = 0.004).^69^ Lisboa and colleagues subsequently benchmarked a self-explaining Partial Response Network against IHTSA and Explainable Boosting Machines on external validation cohorts from OPTN (n = 4750) and a Scandinavian registry, achieving AUCs of 0.628–0.643 across models, all exceeding IMPACT but none substantially surpassing IHTSA.^21^ Zhou et al. built a generalisable interpretable neural network around just nine clinical variables and showed it held up across four geographically distinct cohorts (UNOS, Eurotransplant, Scandiatransplant, and an external Chinese cohort), with external areas under the receiver operating characteristic curve (AUROCs) of 0.827, 0.789, 0.776, and 0.821, respectively.^23^ A 2025 meta-analysis of 12 studies yielded a pooled AUC of 0.65 ((95% confidence interval [CI] 0.64–0.67), confirming a consistent but modest advantage over traditional regression.^22^ Miller et al. found that multiple machine learning models achieved only comparable modest discrimination (C-statistic up to 0.66) to traditional logistic regression on 56,477 OPTN heart transplant recipients.^12^ In a paediatric application, Altamimi et al. applied a random survival forest to 4162 children in the UNOS registry, stratifying graft-failure risk into low- and high-risk groups (1-year survival 92.8% vs 88.1%) albeit with modest discrimination (1-year AUC 0.636; C-index 0.60).^20^ In a larger registry analysis, Dolatsara et al. applied a two-stage framework with isotonic calibration to 103,570 transplants, producing calibrated 1-to-10-year survival probabilities with discrimination of 0.60–0.71.^16^ Kampaktsis et al. achieved an AdaBoost AUC of 0.689 for 1-year mortality in 18,625 recent OPTN transplant recipients, outperforming the IMPACT score (0.569).^14^ Earlier work by Agasthi et al. using gradient boosted machines achieved AUCs of 0.717 for 5-year mortality and 0.716 for graft failure, identifying the top 10 influential variables for risk stratification.^13^ Yoon et al. used a Trees of Predictors approach in 51,971 adult heart transplant recipients to generate individualised survival estimates that outperformed standard clinical risk scores.^17^ In a single-centre Chinese cohort of 381 recipients, a random forest reached an AUC of 0.801 for 1-year mortality, with serum albumin, recipient age, and left atrial diameter as the leading predictors.^18^ In children, Miller et al. compared neural network, decision tree, and random forest models in 2802 paediatric recipients, reaching AUCs of 0.72, 0.61, and 0.60 for 1-, 3-, and 5-year mortality.^19^

For lung transplantation, survival prediction models have generally outperformed their cardiac counterparts, likely because pulmonary physiology variables provide richer discriminative features. Tian et al. reported a random survival forest model trained on 504 Chinese recipients achieving an integrated AUC of 0.879 with internal bootstrap validation, though generalisability to other populations remains unestablished.^40^ Zafar et al. developed a Cox-LASSO tool in 15,124 recipients that stratified risk and predicted 1-, 5-, and 10-year survival, with C-statistics of 0.67, 0.64, and 0.72.^42^ A large-scale study using 29,364 patients from the ISHLT registry reported that gradient boosting achieved an AUC of 0.852 on external validation from a Korean centre, although this represented a notable decrement from the internal validation AUC of 0.958.^41^ By contrast, Dalton et al.^7^ and Brahmbhatt et al.^43^ found that ML approaches applied to the United States Lung Allocation Score (LAS) framework did not improve upon traditional models for post-transplant survival prediction (AUC 0.55 to 0.62), suggesting that available pre-transplant variables may represent an intrinsic information ceiling for certain prediction tasks. This ceiling may partly reflect the design of allocation frameworks such as the CAS, which incorporates up to 5-year post-transplant survival predictions alongside non-clinical factors including prior living organ donation status and donor–recipient proximity that serve broader policy objectives beyond survival prediction alone. Moro et al. combined logistic regression with a survival tree in 27,296 recipients to derive eight prognostic subgroups (C-index 0.653).^44^ Amini et al. used an explanatory analytics framework in 13,080 recipients, with a random forest reaching 77.9% accuracy and surfacing the main drivers of prolonged survival.^45^ While early lung transplant models relied on small cohorts, our group's previously published study (Sharma et al., 2025)^39^ addressed the interpretability gap using the AutoScore-Survival framework on a national cohort of 51,933 recipients from the OPTN registry. Our 9-variable hybrid model achieved good calibration and moderate discrimination (iAUC 0.61) in temporally split testing (2015–2025), demonstrating that parsimonious, interpretable models can maintain predictive utility without the ‘black box’ opacity of complex deep learning approaches. The findings of the accompanying meta-analysis are presented in the Supplementary data.

An important methodological caution emerged from Miller et al., who showed that conventional shuffled cross-validation dramatically overestimates real-world performance.^70^ Using 59,590 adults from UNOS, they demonstrated that random forests achieved an AUC of 0.893 with shuffled cross-validation but only 0.657 with temporally appropriate rolling cross-validation, a finding that mandates more rigorous evaluation strategies across the field. This discrepancy illustrates the danger of data leakage from future observations informing model training, an issue particularly relevant in transplantation where temporal trends in surgical technique, donor management, and immunosuppression protocols shift continuously. Notably, recent robust studies have adopted this temporal validation approach to avoid data leakage, confirming that realistic performance metrics often settle in the 0.60–0.65 range for long-term survival.^39^

### Donor organ evaluation

Effective lung transplant matching requires precise size compatibility. For lung transplantation, sizing relies on computed tomography (CT) volumetry, predicted and measured total lung capacities, and anthropometric parameters (age, sex, height, weight); for heart transplantation, three-dimensional volumetric analysis and echocardiographic and haemodynamic parameters are used. In principle, AI could support these tasks by estimating respiratory compliance from ventilatory data, screening donor CT scans for anatomical anomalies, and refining waitlist prioritisation for candidates supported by ventricular assist devices or extracorporeal membrane oxygenation. In a single-centre series of 404 transplants, Dueñas-Jurado et al. found forced vital capacity and forced expiratory volume in 1 s (FEV1) to be the key donor-recipient matching predictors of 6-month graft survival.^47^ The 2018 UNOS allocation revision dramatically increased the proportion of candidates bridged from temporary mechanical circulatory support (MCS) devices, adding a layer of haemodynamic and device-related complexity that conventional scoring systems were not built to handle.^71^ AI models integrating device type, support duration, and end-organ trajectory could improve pre-transplant risk stratification in this growing population, though externally validated tools for this specific application do not yet exist.

### Waitlist mortality prediction and donor organ assessment

Beyond post-transplant outcomes, ML has been applied to predict waitlist mortality and assess donor organ quality. Hsich et al. applied random survival forests to 33,069 adults from the Scientific Registry of Transplant Recipients (SRTR) database (which provides identical data to the OPTN registry) and identified estimated glomerular filtration rate (eGFR), serum albumin, extracorporeal membrane oxygenation use, and ventricular assist device status as the strongest predictors of heart transplant waitlist death; notably, eGFR and albumin, both routinely available, prognostically important markers of end-organ function and nutritional status, are not formally weighted in the current OPTN heart allocation algorithm, illustrating how a data-driven approach can surface predictors overlooked by expert-designed scoring systems.^24^ Medved et al. earlier applied neural networks to 27,444 waitlisted candidates to predict whether patients would remain waiting, undergo transplantation, or die at 180, 365, and 730 days.^25^ A related simulation by the same group modelled allocation across 49,566 records and projected longer mean survival under neural-network allocation than under wait-time or clinical-rule allocation.^26^

Ram and colleagues developed a dictionary-learning classifier applied to ex-vivo CT scans of 100 donor lung pairs, achieving a classification accuracy of 85% and AUC of 0.89 for predicting transplant suitability.^48^ Recipients of lungs classified as unsuitable by the ML model, but accepted by clinical teams, were 19 times more likely (95% CI 3.98–91.80) to develop chronic lung allograft dysfunction (CLAD) within 2 years. CLAD is the leading cause of late graft loss after lung transplantation, affecting up to 50% of recipients by 5 years, and therapeutic options remain limited once established fibrosis develops, making early identification of at-risk grafts a critical priority. Although the wide confidence interval reflects limited event numbers and should be interpreted cautiously, these findings suggest that algorithmic assessment can identify subclinical injury invisible to conventional evaluation. Sauthier et al. developed a temporal deep neural network for automated identification of potential organ donors from routine intensive care unit data, achieving an AUC of 0.966 and identifying patients missed by both medical teams and manual death audits.^52^ Mark et al. used machine-learning survival curves to show that accepting lungs from an increased-risk donor conferred a 7.2% higher 5-year survival than waiting for a standard organ.^46^ Quantitative modelling has also influenced allocation policy. The OPTN CAS for lung transplantation, implemented in March 2023, was developed using mathematical optimisation of allocation priorities. Early national data indicate that, under the CAS, waitlist mortality fell (sub-hazard ratio 0.79; 95% CI 0.69–0.92) and transplant rates rose, with similar 9-month post-transplant survival.^72^ The CAS itself is not a machine-learning model, although AI/ML analyses informed weight selection during policy design.

## AI in the peri-operative phase

### Ex-vivo lung perfusion and machine learning

The InsighTx platform represents the most mature integration of ML into clinical transplant workflow. Trained on 725 ex-vivo lung perfusion (EVLP) cases using extreme gradient boosting (XGBoost) with physiological, biochemical, and cytokine data, InsighTx achieved areas under the receiver operating characteristic curve (AUROCs) of 0.79 ± 0.03 in training and 0.75 ± 0.04 and 0.85 ± 0.03 in two independent test datasets, with particularly strong performance for identifying unsuitable lungs (AUROC 0.90 ± 0.04).^49^ In a blinded implementation study, InsighTx increased the likelihood of transplanting suitable donor lungs (odds ratio [OR] 13; 95% CI 4–45) while decreasing the likelihood of transplanting unsuitable organs (OR 0.4; 95% CI 0.16–0.98).^49^ In practical terms, decision support shifted real-time acceptance toward suitable organs and away from unsuitable organs, indicating that the model could expand the usable donor pool while guarding against the use of poor-quality lungs. This was the first demonstration that an ML model could safely influence organ utilisation decisions in real time, and it remains the strongest evidence for clinical impact of AI in transplantation. Chao et al. extended this work by applying ResNet-50 convolutional neural networks to 1300 longitudinal EVLP radiographs.^50^ When combined with the InsighTx clinical model, the multimodal system achieved an AUROC of 0.94, representing a significant improvement over the clinical model alone (p < 0.0001). These findings from the Toronto group have established EVLP-based AI as the leading edge of transplant ML, demonstrating that integrating imaging and clinical data can surpass either data source in isolation. Supporting the multimodal approach, Pu et al. developed automated lung volume estimation from CT using U-Net combined with MLP/XGBoost, achieving a Dice coefficient of 0.951 for right lung segmentation and R^2^ of 0.628 for volume prediction, facilitating donor-recipient size matching.^51^

### Prediction of primary graft dysfunction

Primary graft dysfunction (PGD) is the leading cause of early mortality after both heart and lung transplantation. ML approaches to cardiac PGD prediction have progressed from single-centre efforts to international collaborations. The International Consortium on Primary Graft Dysfunction, a multicentre registry across 10 centres in North America and Europe (2746 recipients; 215 [7.8%] with severe PGD), found that the most validated existing tool, the RADIAL score, discriminated poorly in the contemporary era (AUC 0.53, 95% CI 0.48–0.58) and instead identified acute preoperative dialysis, durable left ventricular assist device support, and total ischaemic time as independent risk factors for severe PGD by multivariable mixed-effects logistic regression.^73^ Ding et al. used five ML models on 8008 OPTN transplant recipients and reported that a multi-layer perceptron achieved an AUROC of 0.868, while explainable AI methods identified the top 20 donor–recipient features and their interactions influencing PGD risk.^37^ Most recently, Yim et al. developed an XGBoost-informed nomogram trained on 25,200 OPTN transplant recipients (2008–2020), incorporating eight donor and recipient predictors with an AUC of 0.67 (95% CI 0.64–0.70), externally validated in a Chinese cohort (AUC 0.67), and demonstrated that patients in the highest-risk stratum had a 2.4-fold increased hazard of 90-day graft failure.^38^ For lung PGD, Diamond and colleagues developed a regularised logistic regression model from a prospective multicentre cohort, achieving a C-statistic of 0.76 with decision curve analysis on an external validation cohort demonstrating net benefit across risk thresholds of 10%–75%.^53^ A French single-centre study introduced sequential intraoperative modelling using XGBoost, progressively integrating surgical data across nine chronological steps and reaching a peak AUROC of 0.84 at second graft implantation, demonstrating the value of real-time risk updating during surgery.^55^ Ma et al. recently showed that incorporating donor-lung CT images via computer vision improved PGD prediction beyond electronic health record data alone, establishing a multimodal paradigm that mirrors the clinical process of integrating radiographic and physiological information.^74^ The ability to stratify PGD risk preoperatively and intraoperatively could enable targeted prophylactic strategies, inform resource allocation in intensive care, and support more informed donor organ acceptance decisions.^74^ Additional single-centre studies have contributed to PGD risk modelling. Xia et al. achieved an AUROC of 0.80 using random forests with SHAP interpretation of 9 selected clinical risk factors,^56^ while Michelson et al. developed an open-access risk calculator achieving AUROC of 0.65 with KNN algorithms.^54^ Cantu et al. showed that donor-lung gene expression in innate immune toll-like-receptor pathways predicted primary graft dysfunction (AUC 0.776) in 113 recipients.^57^

## AI in post-transplant rejection surveillance

### Deep learning for endomyocardial biopsy grading

Digital pathology powered by deep learning has achieved the most compelling results in post-transplant AI. The Cardiac Rejection Assessment Neural Estimator (CRANE), published by Lipkova et al., used attention-based multiple-instance learning on gigapixel whole-slide images from endomyocardial biopsies across US, Turkish, and Swiss centres.^28^ CRANE achieved an AUC of 0.962 (95% CI 0.943–0.980) for overall rejection detection, 0.958 for acute cellular rejection, 0.874 for antibody-mediated rejection, and 0.939 for Quilty B lesion identification. In a reader study, AI assistance improved accuracy across all tasks for all pathologists while reducing inter-observer variability and assessment time. These results are particularly notable given the well-documented 20–30% inter-observer variability in endomyocardial biopsy grading even among expert pathologists.^75^ Self-supervised pre-training has further enabled deep learning to achieve AUROCs of 0.849 for binary rejection detection when trained on only 1079 slides from 325 patients across three German centres,^76^ an approach particularly relevant for transplant pathology, where annotated datasets are scarce and expert labelling is resource-intensive. An early proof of concept by Tong et al. applied a deep neural network with dropout to whole-slide endomyocardial biopsies from 43 patients, reducing overfitting relative to unregularised models.^27^ Together, these studies demonstrate that AI-assisted biopsy grading can match or exceed human performance, addressing one of the most established sources of diagnostic variability in transplant medicine.

### Molecular diagnostics and the molecular microscope

The Molecular Microscope Diagnostic System (MMDx) applies unsupervised ML to microarray-based transcript profiling of biopsy tissue.^29^^,^^77^ In 3230 cardiac biopsies (1641 from the INTERHEART study plus 1589 clinical service biopsies), six injury-independent classifiers defined seven rejection archetypes, showing that rejection varies along intensity gradients rather than falling into discrete categories, and that parenchymal injury, more than antibody-mediated rejection, was associated with graft loss. In a separate real-world cohort of 228 paired specimens from 135 recipients, MMDx and histology agreed in 84% of cases (Cohen's kappa 0.48, p < 0.001), with the molecular assay identifying more rejection events than histology alone.^29^^,^^30^^,^^77^^,^^78^ For lung transplantation, MMDx has been applied to 242 transbronchial biopsies, successfully classifying molecular rejection phenotypes that may not be apparent on histological examination.^64^^,^^65^ By detecting molecular rejection and early inflammation that precede histological changes, MMDx can reclassify histologically ambiguous biopsies and, in principle, inform decisions to adjust immunosuppression. These molecular phenotypes, including mixed rejection states, may ultimately redefine the classification of transplant rejection.

### Cell-free DNA and gene expression profiling

Donor-derived cell-free DNA (dd-cfDNA) has emerged as a powerful noninvasive biomarker for allograft injury. For heart transplantation, the donor-derived cell-free DNA (dd-cfDNA) assay (AlloSure) was validated prospectively across 26 US centres, demonstrating 44% sensitivity for acute rejection at a threshold of 0.2%, with a negative predictive value (NPV) exceeding 97%.^31^ For lung transplantation, AlloSure Lung achieved 74% sensitivity, 88% specificity, and 97% NPV, with the potential to reduce invasive surveillance bronchoscopies by 82–83%.^79^ The high NPV is particularly valuable clinically, as a negative result provides confidence that an invasive biopsy is unnecessary, with implications for patient comfort, procedural risk, and resource utilisation. The clinical adoption of dd-cfDNA monitoring is accelerating, with several transplant centres now incorporating these assays into routine surveillance protocols, although the optimal frequency of testing, the interpretation of borderline results, and the cost-effectiveness relative to protocol biopsies remain subjects of ongoing investigation. The combination of dd-cfDNA with gene expression profiling has enhanced diagnostic precision. The SHORE study (67 centres, 2077 heart transplant patients) demonstrated that dual-positive results from the HeartCare platform were associated with a 9.2% incidence of acute cellular rejection, whereas dual-negative results conferred only a 1.5% risk.^80^ Recent prospective multicentre cohorts have refined these estimates. In the 12-centre FreeDNA-CAR trial, Jiménez-Blanco et al. found dd-cfDNA only modestly discriminating for acute cellular rejection alone (AUC 0.60), but adding the routinely available N-terminal pro-B-type natriuretic peptide (NT-proBNP) improved discrimination (AUC 0.68) while preserving the assay's high negative predictive value.^32^ In the GRAfT registry, Mehta et al. showed dd-cfDNA tracked clinical outcomes more closely than histopathological grading, while documenting substantial interrater variability in antibody-mediated rejection scoring.^33^ AlloMap, the first US Food and Drug Administration (FDA)-cleared molecular test for transplant rejection surveillance, was validated in the IMAGE trial, which established that gene expression profiling-guided monitoring was non-inferior to routine endomyocardial biopsy.^81^

### Novel noninvasive approaches

**Fig. 2:**
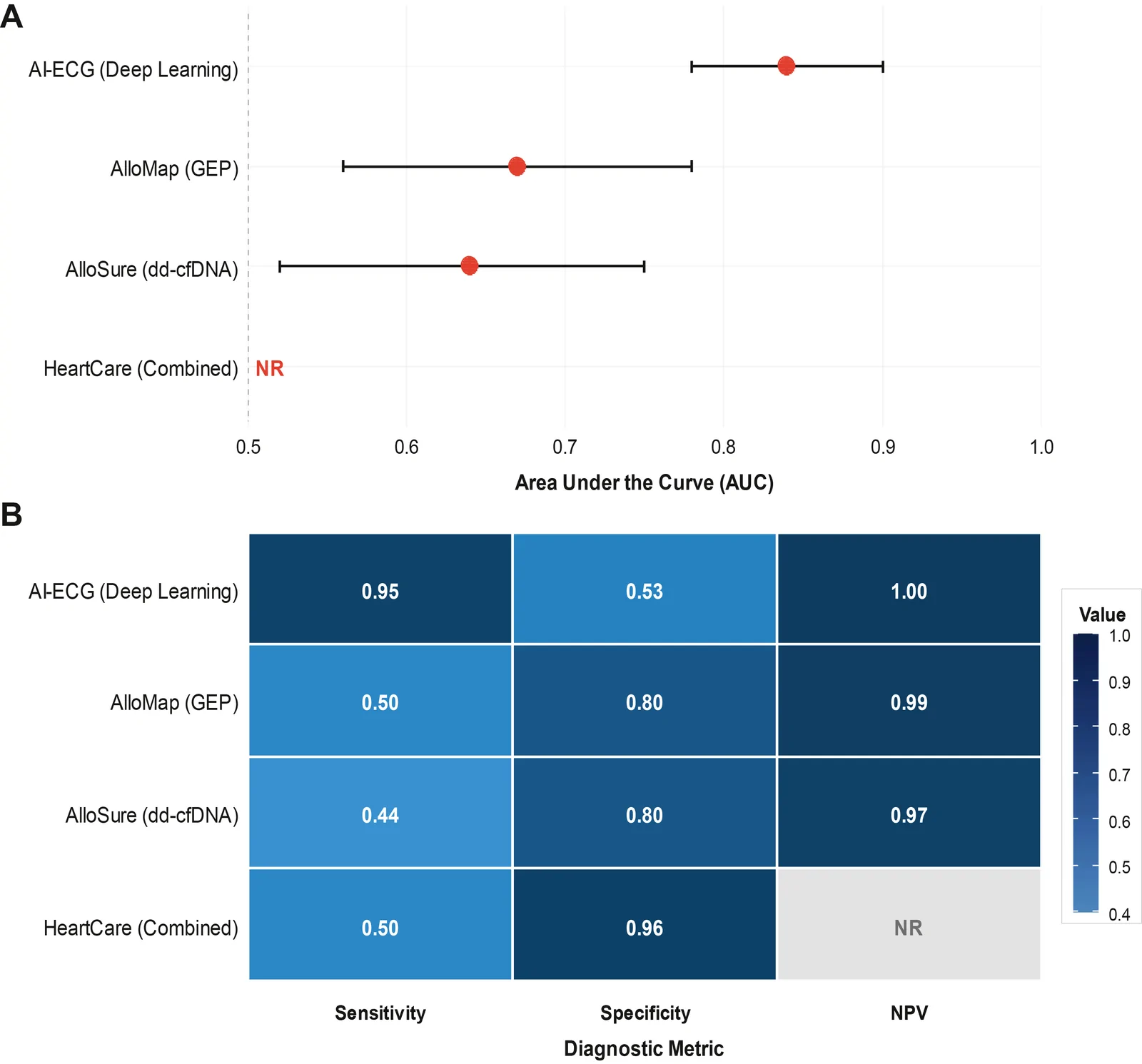
**Comparative diagnostic performance of noninvasive rejection surveillance modalities in heart transplantation**. (A) Area under the curve (AUC) values for combined artificial intelligence deep learning models (AI-ECG), gene expression profiling (AlloMap), and donor-derived cell-free DNA (AlloSure), multianalyte platforms (HeartCare), with error bars representing 95% confidence intervals where available. (B) Corresponding negative predictive value (NPV), sensitivity, and specificity metrics for each diagnostic modality. Data reflect findings from the largest published validation studies for each respective technology. HeartCare NPV is shown as NR; the source reports HeartCare sensitivity and specificity (pooled from two single-centre studies) but not a combined negative predictive value or AUC. NR = not reported, Values are rounded to two decimals. Abbreviations: AI = artificial intelligence; AI-ECG = artificial intelligence-based electrocardiogram model; AUC = area under the curve; CI = confidence interval; dd-cfDNA = donor-derived cell-free DNA; NPV = negative predictive value; NR = not reported.

Adedinsewo et al. developed a convolutional neural network for rejection detection in 12-lead electrocardiograms (ECGs), trained on 7590 ECG–biopsy pairs from 1427 heart transplant recipients.^34^ The AI-ECG model achieved an AUC of 0.84 (95% CI 0.78–0.90) with 95% sensitivity and 99.7% NPV, outperforming conventional ECG analysis (AUC 0.579) and the reported discrimination of gene expression profiling (AUC approximately 0.67) and dd-cfDNA (AUC 0.64) assays. A prospective proof-of-concept study (n = 56) confirmed an AUC of 0.78 with 100% sensitivity. This approach is notable for its simplicity, low cost, and scalability, as standard 12-lead electrocardiography is universally available and incurs negligible marginal expense compared with molecular assays. Burrello et al. demonstrated that random forest analysis of extracellular vesicle surface antigen profiles achieved an AUC of 0.968 in leave-one-out cross-validation, though this performance estimate from only 24 patients is likely optimistic and requires validation in substantially larger cohorts.^35^ Although limited by small sample size (24 patients, 285 samples), this work suggests that liquid biopsy approaches may evolve beyond cfDNA to incorporate broader circulating biomarker panels. By contrast, a large prospective multicentre study evaluating circulating microRNAs for heart transplant rejection surveillance was stopped for futility, tempering enthusiasm for microRNA-based diagnostics and underscoring the importance of prospective validation before clinical adoption (Fig. 2).^82^

### Personalised immunosuppression and pharmacogenomics

Tacrolimus remains a cornerstone of post-transplant immunosuppression, yet its narrow therapeutic index and wide inter-individual pharmacokinetic variability make dosing a persistent clinical challenge. Traditional therapeutic drug monitoring relies on trough levels, but these capture only a single point on the pharmacokinetic curve and often fail to predict area-under-the-curve exposure adequately. The clinical consequences are substantial: subtherapeutic concentrations increase rejection risk, whereas supratherapeutic concentrations increase the risks of nephrotoxicity, infection, and other adverse effects.^83^^,^^84^ Choshi et al. developed a multivariate long short-term memory (LSTM) time-series model for personalised tacrolimus trough level prediction after lung transplantation using mainly prior dose, route, and historical trough data. Trained on 87,112 data points from 117 patients, the model achieved predictions within 30% of actual values at 88.5% of time points and enabled dose–response simulation. The work demonstrates that longitudinal dose–concentration trends alone can support clinically useful forecasting and may help reduce dosing errors without reliance on complex or non-routine variables.^66^ Pharmacogenomic factors contribute substantially to tacrolimus variability. CYP3A5 genotype is consistently identified as the most influential genetic determinant of tacrolimus metabolism, and the Clinical Pharmacogenetics Implementation Consortium guidelines recommend genotype-guided initial dosing.^85^^,^^86^ AI-driven dosing models must also integrate genetic risks like short telomere syndrome, as recent ISHLT guidelines note reduced marrow reserve often requires immunosuppression adjustment because of cytopenias.^87^

## Long-term outcome prediction

### Chronic lung allograft dysfunction

CLAD, including both bronchiolitis obliterans syndrome (BOS) and restrictive allograft syndrome (RAS), remains the principal barrier to long-term lung transplant survival, and accounts for the majority of late graft loss. Early detection of CLAD is essential, as therapeutic options are limited once fibrosis has developed. Barbosa and colleagues demonstrated that support vector machines trained on quantitative CT parameters could distinguish patients who would develop BOS from those who would not, achieving 85% accuracy.^60^ Barbosa et al. earlier reported that multivariate quantitative CT modelling discriminated BOS with AUCs of 0.817 in unilateral and 0.765 in bilateral recipients.^59^ Using the CALIPER CT tool in 88 recipients, McInnis et al. phenotyped CLAD and predicted graft failure (AUC 0.851).^61^ Berra et al. applied a support vector machine to angiotensin II-regulated bronchoalveolar lavage peptides in 40 recipients, identifying established CLAD (AUC 0.86) and predicting future CLAD (AUC 0.97).^62^ Wijbenga et al. combined electronic-nose breath profiling with clinical data in 152 recipients, reaching an AUC of 0.94 for CLAD detection.^63^ A recent study by Kozinski et al. achieved an AUC of 0.90 (95% CI 0.840–0.953) for BOS detection from standard-resolution CT using a deep neural network with a novel co-training strategy designed for low-data scenarios.^58^ The ability to detect early BOS from routine imaging, before spirometric decline becomes apparent, could enable pre-emptive intervention and represents one of the most clinically impactful potential applications of AI in lung transplantation.

### Cardiac allograft vasculopathy

Cardiac allograft vasculopathy (CAV) is a leading cause of late mortality after heart transplantation, characterised by diffuse intimal hyperplasia that is often undetectable by standard coronary angiography until advanced disease has developed. Moayedi et al. applied latent class mixed-effects modelling to serial intravascular ultrasound measurements from 186 recipients, identifying four distinct phenotypic clusters with 5-year risks ranging from 0% to 49.1%.^36^ Separately, for overall post-transplant risk stratification, Ayers et al. demonstrated that ensemble methods combining deep neural networks, random forests, AdaBoost, and logistic regression achieved an AUC of 0.764 for 1-year all-cause mortality, outperforming individual traditional risk models.^15^ This phenotypic stratification enables targeted surveillance intensification for high-risk patients while potentially reducing unnecessary invasive procedures in those at low risk. The application of AI to intravascular imaging is particularly promising because coronary angiography, the current standard surveillance modality, has well-documented insensitivity for the diffuse intimal disease characteristic of CAV. Automated intravascular ultrasound segmentation using deep learning has shown excellent agreement with expert annotation (correlation coefficient 0.98 for lumen area), and automated three-dimensional optical coherence tomography analysis has demonstrated speed and reproducibility unachievable by manual methods.^88^^,^^89^

### Remote monitoring and digital health

Odisho et al. demonstrated that automated chatbot-based home spirometry monitoring achieved a correlation of 0.93 with laboratory-measured FEV1 after lung transplantation, and that novel alert algorithms for clinically significant decline showed promise for early detection of acute respiratory events.^67^ These remote monitoring platforms may be particularly valuable for patients living far from transplant centres, potentially reducing the burden of travel for routine surveillance while maintaining early detection capability. Continuous home monitoring data could detect functional decline before conventional spirometry, and wearable signals (heart rate variability, activity, oxygen saturation) are amenable to ML analysis, although clinical validation of wearable-derived biomarkers in transplant populations remains early.

## Challenges and limitations

### The performance ceiling

A recurring finding across transplant ML studies is the modest magnitude of improvement over traditional methods. The published meta-analytic pooled AUC of 0.65 for heart transplant mortality prediction represents only a modest increment above the C-statistic of 0.61 achieved by the IMPACT logistic regression model.^22^ This finding suggests that ML's apparent superiority in some studies derives from incorporating more variables rather than from algorithmic sophistication per se. Lisboa and colleagues reached a similar conclusion, arguing that the limiting factor in transplant mortality prediction is noise in the underlying data rather than model complexity.^21^ The implication is that better prediction depends primarily on better data (richer phenotyping, molecular characterisation, and real-time physiological monitoring) rather than on more complex algorithms applied to existing registry variables, which should direct research and funding toward prospective, multi-modal data infrastructure. This also has direct implications for sex-disaggregated analysis: performance stratified by sex assigned at birth is inconsistently reported across the transplant ML literature, and this gap limits our ability to assess whether current models serve all patient groups equitably.

### Data scarcity and methodological rigour

Thoracic transplantation is inherently a low-volume discipline, with approximately 5887 hearts and 3771 lungs transplanted annually worldwide, creating datasets far smaller than those typical in ML research.^1^ Registry data, while valuable for scale, often contain substantial missingness, inconsistent coding across centres, and limited granularity compared with prospectively collected cohort data. Critical variables such as intraoperative haemodynamics, detailed echocardiographic parameters, and longitudinal immunosuppressive drug levels are typically absent from registry datasets. Class imbalance presents an additional challenge; rejection events are relatively uncommon in contemporary immunosuppressive regimens, and early mortality has declined over time, creating skewed outcome distributions that can bias model training toward the majority class. Standard discrimination metrics such as AUC may overestimate apparent performance in the presence of extreme class imbalance, and calibration, which reflects the agreement between predicted probabilities and observed event rates, is infrequently reported despite its importance for clinical decision-making.

External validation remains critically deficient; only a minority of published ML prediction models report external validation on geographically or temporally distinct cohorts.^90^ The performance degradation between internal and external validation is striking: the ISHLT lung transplant gradient boosting model dropped from an internal AUC of 0.958 to 0.852 on Korean external data.^41^ Adherence to reporting standards such as the Transparent Reporting of a multivariable prediction model for Individual Prognosis Or Diagnosis for artificial intelligence (TRIPOD + AI), which was updated in 2024 with a 27-item checklist, remains inconsistent across the field.^91^ Without standardised reporting, it is difficult to compare models, identify sources of bias, or assess clinical readiness.

### Algorithmic bias, health equity, and the reporting of race and ethnicity

**Fig. 3:**
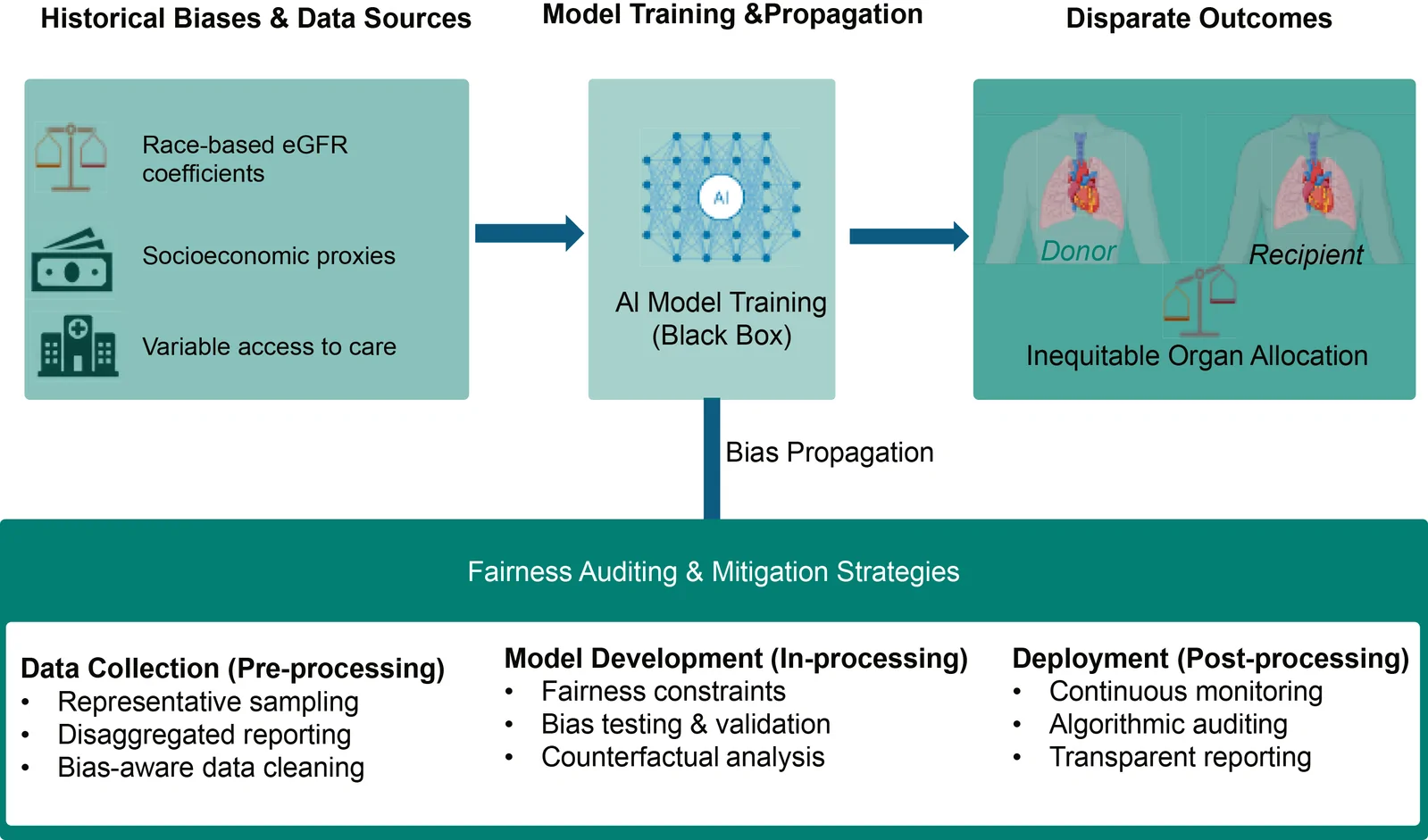
**Algorithmic bias in organ allocation: a conceptual framework for fairness auditing**. Diagram illustrating how historical biases in transplant data (race-based eGFR coefficients, socioeconomic proxies, variable access to care) can propagate through AI model training to produce disparate outcomes in organ allocation. The framework presents recommended mitigation strategies at each stage: data collection (representative sampling, disaggregated reporting), model development (fairness constraints, bias testing), and deployment (continuous monitoring, algorithmic auditing, transparent reporting). Abbreviations: AI = artificial intelligence; eGFR = estimated glomerular filtration rate.

The introduction of AI in organ allocation raises reasonable equity concerns. The historical inclusion of a race coefficient in eGFR formulae systematically overestimated renal function in Black patients.^92^ Although clinical practice no longer uses race-based eGFR formulae, their historical use in assessing renal candidacy for heart transplants impacts thoracic transplant AI development. Models trained on this older data risk perpetuating algorithmic bias, which could lead to inequitable organ allocation and measurable harm. This episode illustrates how algorithms encoding sociocultural constructs as biological variables can produce systematic harm at scale. More broadly, any AI model trained on data reflecting historical inequities in access or diagnostic intensity may reproduce those inequities, even without explicit inclusion of race as a variable (Fig. 3).

Because race is a sociocultural construct rather than a fixed biological trait, race-based algorithms risk encoding structural inequities into clinical decision tools. AI systems trained on historically biased data may perpetuate or amplify disparities through proxy variables that correlate with race, including postcode, insurance status, and comorbidity burden.^93^ Algorithmic fairness frameworks have been cited in the transplant ML literature but rarely embedded at the model development stage.^94^ Continuous bias auditing, representative training datasets, and algorithmic fairness constraints must be integral to any AI system deployed in organ allocation. Researchers developing transplant AI tools should plan to report and analyse model performance stratified by race, ethnicity, sex, and socioeconomic status to help uncover health inequities, in accordance with current reporting recommendations. Most of the models reviewed here were developed in registry cohorts from North America and Europe, so the populations underpinning current transplant AI are not representative of the global transplant population and minoritised racial and ethnic groups are variably represented within them. Any race-associated differences in model performance should therefore be interpreted alongside socioeconomic position, referral and listing patterns, and other structural drivers that are seldom captured in registry data, rather than as intrinsic biological differences. Transparent, disaggregated reporting offers a practical opportunity to direct model refinement and resources toward the groups that existing allocation tools have served least well.

### Explainability and regulatory landscape

The opacity of many ML models presents a fundamental challenge in transplant medicine, where decisions directly affect survival and where clinician trust is essential for adoption (Supplementary Table S1).^10^^,^^95^ Transplant clinicians are accustomed to transparent decision frameworks in which the influence of each variable is understood, and the introduction of opaque algorithmic recommendations into this context raises questions about accountability, particularly when model outputs conflict with clinical judgement.^95^ Post-hoc explainability methods, including Shapley Additive Explanations (SHAP) and Local Interpretable Model-Agnostic Explanations (LIME), have been applied in several transplant studies, but these provide approximations of model reasoning rather than true mechanistic insight. Intrinsically interpretable models may be preferable for high-stakes allocation decisions, even at the cost of marginal performance decrements. To our knowledge, none of the AI/ML tools reviewed here for transplant allocation or organ-acceptance decision-making has received FDA marketing authorisation or CE marking for that indication. The FDA's AI/ML Software as a Medical Device Action Plan introduced a Total Product Lifecycle approach for continuously learning algorithms, with Predetermined Change Control Plan guidance finalised in December 2024, which may be particularly relevant for transplant models operating in evolving clinical environments; to date, authorised AI and ML medical devices have concentrated in a small number of specialities such as radiology rather than transplantation.^96^ Under the European Union’s AI Act, medical-device AI systems that require third-party conformity assessment are generally classified as high-risk.^97^

Beyond algorithmic bias in model design, the practical deployment of AI in transplantation raises important concerns about global health equity and resource distribution. High-performing AI systems typically depend on computational infrastructure, digitised pathology workflows, high-throughput molecular assays, and large curated datasets. These resources are concentrated in high-income transplant centres, many of which generated the training data that underpin current models. For transplant programmes in low- and middle-income countries (LMICs), where organ donation rates are also constrained by regulatory, logistical, and cultural barriers, this infrastructure gap represents a substantive implementation ceiling. The risk is that advances in AI may widen rather than narrow global disparities in transplant outcomes, accelerating progress at well-resourced centres while remaining inaccessible in settings where the burden of end-stage cardiac and pulmonary disease is equally severe. Future transplant AI development must actively account for LMIC contexts through locally adaptable models, lower-resource diagnostic platforms such as point-of-care biomarker assays, and collaborative research partnerships that treat equity as a design requirement rather than an afterthought.

### Reporting limitations and patient perspectives

A formal risk-of-bias assessment using the Prediction model Risk Of Bias Assessment Tool (PROBAST) was not conducted for individual studies; this is acknowledged as a limitation of the evidence synthesis, and readers should interpret pooled AUC estimates as descriptive summaries rather than precise performance projections. Furthermore, patient perspectives on AI-assisted transplant decision-making represent an important dimension that this Review has not addressed in depth. Recipients navigate prolonged uncertainty throughout the transplant journey, from waitlist listing through organ acceptance to long-term surveillance, and the introduction of algorithmic recommendations into these conversations raises meaningful questions about transparency, trust, and informed consent. The limited published evidence suggests that patient attitudes toward AI in high-stakes clinical decisions are variable and context-dependent, shaped by prior experiences, communication quality, and the perceived explainability of recommendations. A full examination of patient acceptability falls outside the scope of this Review, but the transplant community should recognise that technical performance metrics alone do not determine whether an AI system is suitable for clinical use. Future prospective trials should incorporate patient-reported outcomes alongside clinical endpoints, and implementation research should assess how AI recommendations are communicated to patients and how they affect the experience of transplant care.

## Future directions

Federated learning, in which ML models are trained across institutions by exchanging only model parameters rather than raw patient data, offers a privacy-preserving solution to data fragmentation in transplantation.^98^ Sheller et al. demonstrated that federated models trained across 10 institutions achieved 99% of the performance of models trained on centrally pooled data.^68^ Given the multi-centre nature of transplant registries and the sensitivity of transplant data, federated approaches are particularly well suited to this field and could enable effective model development without the regulatory and logistical barriers of centralised data sharing. The digital twin paradigm, creating personalised computational models that simulate treatment responses, has been proposed for thoracic organ transplantation to improve precision medicine, with early foundational work suggesting its feasibility for complex immunological modelling. Multi-omics integration combining genomic, transcriptomic, proteomic, and metabolomic data with clinical variables through AI represents a frontier for precision transplant medicine.^99^ Pre-transplant transcriptomic profiling of peripheral blood mononuclear cells has identified gene expression signatures predictive of rejection, suggesting that pre-operative risk stratification could be enhanced by incorporating molecular data beyond standard clinical variables. Reinforcement learning has been proposed for immunosuppression optimisation, although the exploration–exploitation trade-off, in which the algorithm must occasionally deviate from apparently optimal therapy to learn, raises ethical challenges that distinguish this application from standard clinical trials.

## Conclusions

AI and ML have demonstrated clear potential across the transplant continuum. The most developed applications include deep-learning histopathological grading of cardiac rejection (CRANE: AUC 0.962),^28^ ML-augmented EVLP assessment (InsighTx: AUROC up to 0.90),^49^ noninvasive surveillance with AI-ECG (AUC 0.84)^34^ and dd-cfDNA (NPV >97%),^31^ and CT-based early detection of BOS (AUC 0.90).^58^ Prediction of 1-year post-transplant survival/mortality remains more modest. Across published models, external validation is uncommon, while recalibration to contemporary populations and formal assessment of clinical utility are rarely performed.^21^^,^^22^ Consequently, many published models lack evidence of transportability across settings or demonstrable benefit in guiding clinical decisions. Furthermore, no transplant-specific AI system has yet achieved widespread clinical adoption through robust prospective evaluation, and concerns regarding transparency, algorithmic bias, fairness, and data privacy remain unresolved. Progress will require federated learning across centres, prospective trials of AI-assisted decisions, adherence to TRIPOD + AI, fairness auditing from model inception, and, above all, investment in richer phenotyping and prospective data rather than in more complex algorithms applied to static registry variables.

## Contributors

GS conceptualised the study. GS and VKK conducted the initial literature review and drafted the first version of the manuscript. VKK and GS performed meta-analysis and performance metrics synthesis, and GS and VKK accessed and verified the underlying data (the aggregated statistics extracted from the published studies; no individual participant data were accessed). LT, MF, IT, VK, JM, SK, MP, and MEJ contributed to writing of the clinical content, and SL contributed registry and data-interpretation expertise. All authors participated in the literature review, writing, and critical revision of the manuscript. GS and MEJ were responsible for the decision to submit the manuscript. All authors read and approved the final version of the manuscript.

## Data sharing statement

No original data were collected for this Review. All data referenced are available from the cited published sources.

## Declaration of interests

We declare no competing interests.
