# Supplementary material for: Artificial intelligence and machine learning in heart and lung transplantation
Source: eClinicalMedicine. 2026 Sep 3;99:104180. doi: 10.1016/j.eclinm.2026.104180 (PMC13571553; doi:10.1016/j.eclinm.2026.104180)
Supplement: Supplementary file 1 [file mmc1.docx]

**Supplementary Appendix**

**Artificial intelligence and machine learning in heart and lung transplantation**

Gaurav Sharma, PhD^,2,3,#,^ Vineet Kumar Kamal, PhD^4^, Lauren K. Truby, MD^5^, Maryjane Farr, MD^5,6^, Irina Timofte, MD^7^, Vaidehi Kaza, MD^7^, Srdjan Lesaja, PhD^8^, John SK Murala, MD^1^, Suresh Keshavamurthy, MD^1^, Matthias Peltz, MD^1^, Michael E. Jessen, MD^1#^

From the **^1^**Department of Cardiovascular and Thoracic Surgery, University of Texas Southwestern Medical Center, Dallas, TX, USA; **^2^**Advanced Imaging Research Center, University of Texas Southwestern Medical Center, Dallas, TX, USA; **^3^**Department of Biomedical Engineering, University of Texas Southwestern Medical Center, Dallas, TX, USA; **^4^**Department of Biostatistics, All India Institute of Medical Sciences, Kalyani, India; **^5^**Department of Medicine, Division of Cardiology, University of Texas Southwestern Medical Center, Dallas, TX, USA; **^6^**Penn Heart and Vascular Center, University of Pennsylvania Health System, Philadelphia, PA, United States; **^7^**Department of Internal Medicine, Division of Pulmonary and Critical Care Medicine, University of Texas Southwestern Medical Center, Dallas, TX, USA; **^8^**Department of Research, United Network for Organ Sharing, Richmond, VA, USA

**^#^Correspondence to:**

Dr. Gaurav Sharma, PhD

*Assistant Professor*

Department of Cardiovascular and Thoracic Surgery

Department of Bioengineering Advanced Imaging Research Center

University of Texas Southwestern Medical Center

Dallas, TX 75390, USA

Email: [gaurav.sharma@utsouthwestern.edu](mailto:gaurav.sharma@utsouthwestern.edu)

Prof. Michael E. Jessen, M.D.

*Professor and Chair*

Department of Cardiovascular and Thoracic Surgery

*Frank M. Ryburn, Jr., Distinguished Chair in Cardiothoracic Surgery and Transplantation* University of Texas Southwestern Medical Center

Dallas, TX 75390, USA

Email: [michael.jessen@utsouthwestern.edu](mailto:michael.jessen@utsouthwestern.edu)

**Contents of this supplementary appendix**

S1 Supplementary Methods

S1.1 Meta-analysis statistical methods and reproducibility

S1.2 Study quality and risk-of-bias assessment

S1.3 Protocol registration and reporting statements

S1.4 Patient and public involvement

S1.5 Implementation expertise and workflow integration

S1.6 Findings of the accompanying meta-analysis

Supplementary Figure S1. PRISMA 2020 flow diagram for the embedded meta-analysis of machine-learning models predicting 1-year post-transplant mortality.

Supplementary Figure S2. PRISMA extension for Scoping Reviews (PRISMA-ScR) flow diagram for the Review

Supplementary Figure S3. Forest plots of machine-learning model discrimination for 1-year post-transplant mortality

Table S1. Recommended checklist for reporting AI and ML studies in transplantation

List of Abbreviations

**S1. Supplementary Methods**

**S1.1 Meta-analysis statistical methods and reproducibility**

The meta-analysis presented in Supplementary Figure S3 pooled area-under-the-curve (AUC) statistics from studies reporting machine learning model performance for 1-year post-transplant mortality in separately identified heart transplant and lung transplant cohorts. Literature searches were performed independently for each organ type. Summary AUC values and 95% confidence intervals (CIs), if available, were extracted directly from external validation cohorts. When external validation was unavailable, estimates from independent temporal or held-out testing cohorts were used. Internal cross-validation results were considered only when no separate validation cohort was reported. Where multiple models using different algorithms were evaluated within a study, the best-performing model on the development data was selected. For studies reporting only point estimates of discrimination, standard errors and 95% CIs were derived from the sample size available.

Random-effects meta-analysis was performed using restricted maximum likelihood (REML) estimation to account for between-study variability arising from differences in study populations. The Hartung–Knapp adjustment was applied to obtain more robust confidence intervals for the pooled estimates. Subgroup analyses were performed according to outcome type (binary outcomes versus time-to-event outcomes) for heart transplant models. Forest plots were generated to display study-specific and pooled discrimination estimates with corresponding 95% confidence intervals. Heterogeneity was quantified using the I-squared statistic. A random-effects prediction interval is presented in Supplementary Figure S3 to convey the expected range of performance for a new study drawn from the same population of research settings. Prediction intervals were calculated when at least three studies were available within a meta-analysis. The meta-analysis was conducted using Stata version 18.0 (StataCorp LLC, College Station, TX, USA). Funnel plot assessment and Egger's test for small-study effects were not performed given the small number of studies per analysis (five heart transplant; four lung transplant), which renders these tests statistically underpowered and potentially misleading. This is acknowledged as a limitation. Five heart transplant studies contributed to the pooled heart estimate (where 'n' denotes the specific validation or hold-out testing cohort size utilized for statistical pooling): Miller PE et al. 2019 (n = 17,096), Miller R et al. 2019 (n = 635), Lisboa et al. 2022 (n = 4,750), Zhou et al. 2021 (n = 114), and Nilsson et al. 2015 (n = 1,194). Four lung transplant studies contributed to the pooled lung estimate (where 'n' denotes the specific hold-out testing or validation cohort size utilized for statistical pooling): Sharma et al. 2025 (n = 25,251), Yeo et al. 2025 (n = 228), Zafar et al. 2022 (n = 4,464), and Brahmbhatt et al. 2022 (n = 2,985). The overall pooled AUC was 0.68 (95% CI 0.59 to 0.77) for heart transplantation and 0.69 (95% CI 0.51 to 0.86) for lung transplantation, with wide prediction intervals reflecting extreme between-study heterogeneity (heart I-squared = 93.5%; lung I-squared = 96.6%).

Missing data handling for the meta-analysis: For studies in which confidence intervals were unavailable, standard errors were approximated from sample size, these studies were retained in the meta-analysis to maximize the available evidence while accounting for their uncertainty through inverse-variance weighting. Pooled estimates should be interpreted with caution because the precision of individual studies may not have been fully captured.

**S1.2 Study quality and risk-of-bias assessment**

This is a scoping review with an embedded meta-analysis. A formal individual-study risk-of-bias assessment using the PROBAST tool (Prediction model Risk Of Bias ASsessment Tool) was not conducted. PROBAST assesses four domains: participants, predictors, outcome, and analysis. The Challenges section of the main text addresses methodological concerns across these domains at a narrative level, drawing on patterns observed across the reviewed literature rather than scoring each paper individually. The authors acknowledge that the absence of a systematic PROBAST assessment is a limitation, particularly for the meta-analysis component. The extreme between-study heterogeneity in the meta-analyses (I-squared of 93.5% for heart and 96.6% for lung) is itself a signal that study populations, predictor sets, validation approaches, and outcome definitions vary substantially. Readers should interpret the pooled AUC estimates as descriptive summaries of the published performance range rather than as precise estimates of expected real-world accuracy.

**S1.3 Protocol registration and reporting statements**

**Protocol registration:** This scoping review with an embedded meta-analysis was not prospectively registered. The meta-analysis was conducted using aggregated statistics extracted from publicly available published manuscripts; no individual participant data were accessed or analysed. A formal PROSPERO or OSF registration was not completed prior to the search. The authors acknowledge that prospective registration is best practice for systematic reviews and meta-analyses and recommend this for future reviews in the field.

**Sex and Gender Reporting:** In accordance with eClinicalMedicine author guidelines and the SAGER guidelines, the manuscript addresses sex and gender dimensions of AI in transplantation. The Challenges section includes a statement that model performance stratified by sex assigned at birth is inconsistently reported across the transplant ML literature and that this gap limits the ability to assess whether current models serve all patient groups equitably. The term "sex assigned at birth" is used rather than "biological sex" or "birth sex" throughout. The review acknowledges that most registry datasets do not separately capture gender identity, and that this is a limitation of the underlying evidence base rather than an omission in the review methodology.

**Race and Ethnicity Reporting:** The manuscript discusses race and ethnicity in the context of algorithmic fairness. The algorithmic bias section explicitly states that race is a sociocultural construct rather than a fixed biological trait and explains how race-based eGFR coefficients in historical training data can propagate bias through algorithmic systems. The review avoids race-based reference ranges and algorithmic recommendations, and directs researchers to analyse and report model performance stratified by race and ethnicity to uncover health inequities, in alignment with journal guidance. Where race-associated outcome differences are discussed, the broader structural, socioeconomic, and historical drivers are contextualised.

**Global Health Equity and LMIC Considerations**: The Challenges section includes a dedicated paragraph on this topic, noting that high-performing AI systems depend on computational infrastructure, digitised pathology workflows, high-throughput molecular assays, and large curated datasets that are concentrated in high-income transplant centres. The section explicitly states that the risk exists that advances in AI may widen rather than narrow global disparities in transplant outcomes, and calls for locally adaptable models, lower-resource diagnostic platforms, and collaborative research partnerships that treat equity as a design requirement rather than an afterthought.

**S1.4 Patient and public involvement**

Patients and members of the public were not formally involved in the design, conduct, or reporting of this review. The Review team recognises this as a limitation. Heart and lung transplant recipients navigate a prolonged and often uncertain journey from waitlist listing through organ acceptance, surgery, and lifelong post-transplant surveillance. The introduction of algorithmic recommendations into this pathway raises genuine questions about transparency, trust, and informed consent that go beyond technical performance metrics. Published evidence on transplant patient attitudes toward AI-assisted decision-making is limited but suggests that acceptability is shaped by the perceived explainability of recommendations, communication quality between clinician and patient, and prior experiences of the healthcare system. Patients from minority groups and those with limited health literacy may have additional concerns related to algorithmic fairness that are not yet reflected in most published transplant AI studies.

Future prospective trials evaluating AI-assisted transplant decisions should incorporate patient-reported outcomes as co-primary or secondary endpoints alongside clinical metrics. Implementation research should assess how AI recommendations are communicated to patients, whether patients have meaningful opportunity to understand or contest algorithmic outputs, and how AI integration affects the overall experience of transplant care. The transplant community has an obligation to ensure that patient perspectives inform both the design and deployment of these tools.

**S1.5 Implementation expertise and workflow integration**

The reviewed AI tools vary considerably in the technical infrastructure and clinical expertise required for real-world deployment. The InsighTx EVLP platform requires integration with ex-vivo lung perfusion circuit data streams and real-time model inference during an active surgical preparation, which demands biomedical engineering support alongside transplant surgical and perfusionist expertise. The AI-ECG rejection detection model requires a digitised 12-lead ECG capture and analysis pipeline connected to a transplant cardiologist review workflow. The CRANE digital pathology system for endomyocardial biopsy grading requires whole-slide imaging hardware, a digital pathology informatics infrastructure, and a system for integrating AI-assisted reads with pathologist sign-out workflows.

None of the reviewed studies systematically assessed the implementation burden, clinician trust calibration, or workflow impact of their respective AI tools. Studies reporting clinical integration (InsighTx, AI-ECG proof of concept) provided limited data on how AI outputs were communicated to clinicians, how often outputs were overridden, and what the downstream consequences of disagreement were. These data are essential for understanding whether AI tools function as effective decision support in practice or simply as additional data outputs that clinicians ignore. We recommend that future implementation studies in transplant AI adopt a structured assessment framework, such as the Non-Adoption, Abandonment, Scale-up, Spread, and Sustainability (NASSS) framework, to characterise the organisational, technical, and clinical conditions required for sustained adoption. Prospective randomised trials comparing AI-assisted decision-making versus standard care should specify clinician training requirements, the format and timing of AI output presentation, and escalation pathways when model confidence is low or when clinical judgement conflicts with model output.

**S1.6 Findings of the accompanying meta-analysis**

Nine studies met the criteria for quantitative pooling, five in heart transplantation and four in lung transplantation. Using random-effects models with restricted maximum likelihood estimation and the Hartung-Knapp adjustment, the pooled area under the curve (AUC) for machine learning models predicting 1-year post-transplant mortality was 0.68 (95% confidence interval [CI] 0.59 to 0.77) for heart transplantation and 0.69 (95% CI 0.51 to 0.86) for lung transplantation (Supplementary Figure S3). Between-study heterogeneity was extreme in both analyses, with an I-squared of 93.5% for heart transplantation and 96.6% for lung transplantation.

These pooled estimates should be read as descriptive summaries of the published performance range rather than as precise projections of real-world accuracy. The small number of contributing studies, the absence of a formal individual-study risk-of-bias assessment (Supplementary Methods S1.2), and the substantial heterogeneity in study populations, predictor sets, validation strategies, and outcome definitions all limit interpretability. Prediction intervals, rather than confidence intervals alone, give a more realistic indication of the discrimination that might be expected in a new setting. The magnitude of the pooled estimates is consistent with the narrative synthesis in the main text, in which machine learning models for post-transplant mortality prediction offer only modest gains over established regression-based risk scores.

**Identification of studies via databases and registers**

Records removed *before screening*:

Duplicate records removed

(n = 983)

Records marked as ineligible by automation tools (n = 0)

Records removed for other reasons (n = 0)

Records identified from:

Databases (PubMed 342; MEDLINE 357; Embase 831; Web of Science 435), n = 1,965

**Identification**

Records screened

(n = 982)

Records excluded

(n = 863)

Reports sought for retrieval

(n = 119)

Reports not retrieved

(n = 7)

**Screening**

Reports excluded:103 103

Not a post-transplant mortality prediction model, n = 88

Mortality model, internal cross-validation only (no held-out test set), n = 7

No extractable discrimination metric with variance, n =6

Overlapping cohort with an included study, n = 2

Reports assessed for eligibility

(n = 112)

Studies included in quantitative synthesis (meta-analysis): n = 9 Heart (n = 5): Miller PE 2019; Miller R 2019; Lisboa 2022; Zhou 2021; Nilsson 2015

Lung (n = 4): Sharma 2025; Yeo 2025; Zafar 2022; Brahmbhatt 2022

Pooled AUC: heart 0.68 (95% CI 0.59 to 0.77), I-squared 93.5%; lung 0.69 (95% CI 0.51 to 0.86), I-squared 96.6%. The 9 studies are a subset of the 62 sources charted in the Review

**Included**

**Supplementary Figure S1.** PRISMA 2020 flow diagram for the embedded meta-analysis of machine-learning models predicting 1-year post-transplant mortality. Records were identified from four databases (PubMed, MEDLINE, Embase, Web of Science) for January 1, 2015 to May 31, 2026. Identification and duplicate-removal counts are from the database exports; PubMed and MEDLINE overlap substantially, and de-duplication was performed across all four databases. Reporting standard: Page MJ, et al. BMJ 2021;372:n71.

**Identification of studies via databases and registers**

Records removed *before screening*:

Duplicate records removed, n = 983

Other removals (automation, ineligible), n = 0

Records identified from:

Databases ((PubMed 342; MEDLINE 357; Embase 831; Web of Science 435), n = 1,965

**Identification**

Records screened

n = 982

Records excluded (title and abstract)

n = 863

Reports sought for retrieval

n = 119

Reports not retrieved

n = 7

**Screening**

Reports excluded, with reasons: 28

Not AI or machine learning in heart or lung transplantation, n = 12

No original data (editorial, commentary, narrative review), n = 8

Application or outcome outside the scope of the Review, n = 6

Superseded or duplicate report of an included source, n = 2

Reports assessed for eligibility

n = 112

Studies included in review

(n =84)

Reports of included studies:

Charted by domain across the transplant continuum: donor and organ assessment; ex-vivo perfusion; waitlist and allocation; survival and matching; primary graft dysfunction; rejection surveillance (histopathology, molecular, cell-free DNA, AI-ECG); chronic allograft dysfunction and remote monitoring; immunosuppression.

Of these, 9 mortality-prediction studies met the quantitative criteria and entered the embedded meta-analysis

**Included**

**Supplementary Figure S2.** PRISMA extension for Scoping Reviews (PRISMA-ScR) flow diagram for the Review as a whole. Terminology follows the standard (sources of evidence, charting; no risk-of-bias-across-sources stage). Reporting standard: Tricco AC, et al. Ann Intern Med 2018;169:467-473.

**Supplementary Figure S3.** Forest plots of area under the curve (AUC) values for the best-performing machine learning models predicting 1-year post-transplant mortality in validation cohorts. (A) Heart transplantation. (B) Lung transplantation. Squares represent study-specific AUC point estimates, with square size proportional to study weight; horizontal lines represent 95% confidence intervals (CIs). Diamonds represent the random-effects pooled estimate, with diamond width corresponding to the 95% CI of the pooled estimate. Using random-effects models, the pooled AUC was 0.68 (95% CI 0.59 to 0.77) across five heart transplant studies and 0.69 (95% CI 0.51 to 0.86) across four lung transplant studies. Study weights, 95% CIs, and heterogeneity statistics are presented for each outcome. No box plots, whiskers, or p values are presented. AUC=area under the curve. CI=confidence interval.

| **Domain** | **Recommendation** |
| --- | --- |
| Study design | Adhere to TRIPOD+AI reporting checklist; specify intended clinical use and target population |
| Validation strategy | Include external validation on geographically or temporally distinct cohorts; avoid shuffled cross-validation for time-series data |
| Performance metrics | Report AUC, calibration, sensitivity, specificity, and NPV; include confidence intervals for all metrics |
| Fairness and equity | Report model performance stratified by race, ethnicity, sex, and socioeconomic status; test for differential performance across subgroups |
| Explainability | Provide feature importance rankings and local explanations (SHAP or equivalent); consider intrinsically interpretable models for high-stakes decisions |
| Race and ethnicity | Avoid race-based algorithms; discuss structural determinants when analysing race-associated outcome differences; disaggregate data to uncover inequities |
| Sex and gender | Report participant sex assigned at birth and gender identity where collected; analyse model performance by sex; discuss limitations of data granularity |
| Reproducibility | Share code, model architecture, hyperparameters, and, where possible, de-identified training data; register study protocols prospectively |
| Regulatory readiness | Document conformity with FDA SaMD or EU AI Act requirements; specify Predetermined Change Control Plans for adaptive algorithms |
| Clinical integration | Design prospective trials comparing AI-assisted versus standard clinical decision-making; assess clinician trust and workflow impact |

**Table S1. Recommended checklist for reporting AI and ML studies in transplantation** *AI=artificial intelligence. FDA=Food and Drug Administration. ML=machine learning. NPV=negative predictive value. SaMD=Software as a Medical Device. SHAP=Shapley Additive Explanations. TRIPOD+AI=Transparent Reporting of a Multivariable Prediction Model for Individual Prognosis or Diagnosis updated for AI.*

**List of Abbreviations**

Abbreviations are listed alphabetically. All abbreviations are defined at first use in the main manuscript text or in this appendix.

| **Abbreviation** | **Definition** |
| --- | --- |
| AI | Artificial intelligence |
| AUC / AUROC | Area under the (receiver operating characteristic) curve |
| BAL | Bronchoalveolar lavage |
| BOS | Bronchiolitis obliterans syndrome |
| CAS | Composite allocation score |
| CAV | Cardiac allograft vasculopathy |
| cfDNA | Cell-free DNA |
| CLAD | Chronic lung allograft dysfunction |
| CNN | Convolutional neural network |
| CRANE | Cardiac Rejection Assessment Neural Estimator |
| CT | Computed tomography |
| CYP3A5 | Cytochrome P450 3A5 (gene) |
| DCA | Decision curve analysis |
| dd-cfDNA | Donor-derived cell-free DNA |
| DL | Deep learning |
| ECG | Electrocardiogram |
| eGFR | Estimated glomerular filtration rate |
| EMB | Endomyocardial biopsy |
| EVLP | Ex-vivo lung perfusion |
| FDA | US Food and Drug Administration |
| GBM | Gradient boosted machine |
| iAUC | Integrated area under the curve |
| IHTSA | International Heart Transplant Survival Algorithm |
| IMPACT | Index for Mortality Prediction After Cardiac Transplantation |
| ISHLT | International Society for Heart and Lung Transplantation |
| IVUS | Intravascular ultrasound |
| KNN | K-nearest neighbours |
| LAS | Lung allocation score |
| LASSO | Least absolute shrinkage and selection operator |
| LIME | Local Interpretable Model-Agnostic Explanations |
| LMIC | Low- and middle-income country |
| LR | Logistic regression |
| LSTM | Long short-term memory |
| ML | Machine learning |
| MLP | Multi-layer perceptron |
| MMDx | Molecular Microscope Diagnostic System |
| MRI | Magnetic resonance imaging |
| NN | Neural network |
| NPV | Negative predictive value |
| OPTN | Organ Procurement and Transplantation Network |
| PGD | Primary graft dysfunction |
| PPI | Patient and public involvement |
| RAS | Restrictive allograft syndrome |
| RF | Random forest |
| RSF | Random survival forest |
| SAGER | Sex and Gender Equity in Research |
| SaMD | Software as a Medical Device |
| SHAP | Shapley Additive Explanations |
| SRTR | Scientific Registry of Transplant Recipients |
| SVM | Support vector machine |
| TRIPOD+AI | Transparent Reporting of a Multivariable Prediction Model for Individual Prognosis or Diagnosis, updated for AI |
| UNOS | United Network for Organ Sharing |
| XGBoost | Extreme gradient boosting |
